# Functional Mechanical Behavior and Biocompatible Characteristics of Graphene-Coated Cardiovascular Stents

**DOI:** 10.3390/ijms252413345

**Published:** 2024-12-12

**Authors:** Łukasz Wasyluk, Dariusz Hreniak, Vitalii Boiko, Beata Sobieszczańska, Emanuela Bologna, Massimiliano Zingales, Robert Pasławski, Jacek Arkowski, Przemysław Sareło, Magdalena Wawrzyńska

**Affiliations:** 1 Division of Optical Spectroscopy, Institute of Low Temperature and Structure Research, Polish Academy of Sciences, Okólna 2, 50-422 Wrocław, Poland; l.wasyluk@intibs.pl (Ł.W.); d.hreniak@intibs.pl (D.H.); 2 Carbonmed Ltd., Okólna 2, 50-422 Wrocław, Poland; 3 Institute of Physics, National Academy of Science of Ukraine, Prospect Nauky 46, 03028 Kyiv, Ukraine; 4 Department of Microbiology, Wrocław Medical University, Chałubińskiego 4, 50-368 Wrocław, Poland; beata.sobieszczanska@umw.edu.pl; 5 Department of Engineering, University of Palermo, Viale delle Scienze Ed. 8, 90128 Palermo, Italy; emanuela.bologna@unipa.it (E.B.); massimiliano.zingales@unipa.it (M.Z.); 6 Advanced Technologies Network Center, University of Palermo, Viale delle Scienze Ed. 18/A, 90133 Palermo, Italy; 7 Department of Veterinary Surgery, Institute of Veterinary Medicine, Faculty of Biological and Veterinary Sciences, Nicolaus Copernicus University, Gagarina 7, 87-100 Toruń, Poland; r.paslawski@umk.pl; 8 Pre-Clinical Research Centre, Wrocław Medical University, Marcinkowskiego 1, 50-368 Wrocław, Poland; jacek.arkowski@umw.edu.pl (J.A.); przemyslaw.sarelo@pwr.edu.pl (P.S.); magdalena.wawrzynska@umw.edu.pl (M.W.); 9 Department of Biomedical Engineering, Faculty of Fundamental Problems of Technology, Wrocław University of Science and Technology, Wybrzeże Wyspiańskiego 27, 50-370 Wrocław, Poland

**Keywords:** cardiovascular stent, graphene, cold-wall chemical vapor deposition (CW-CVD), mechanical behavior, endothelialization, biocompatibility

## Abstract

Percutaneous Coronary Intervention (PCI) is a treatment method that involves reopening narrowed arteries with a balloon catheter that delivers a cylindrical, mesh-shaped implant device to the site of the stenosis. Currently, by applying a coating to a bare metal stent (BMS) surface to improve biocompatibility, the main risks after PCI, such as restenosis and thrombosis, are reduced while maintaining the basic requirements for the mechanical behavior of the stent itself. In this work, for the first time, the development and optimization process of the spatial structure of the Co-Cr stent (L-605) with a graphene-based coating using cold-wall chemical vapor deposition (CW-CVD) to ensure uniform coverage of the implant was attempted. The CW-CVD process allows the coating of 3D structures, minimizing thermal stress on the surrounding equipment and allowing the deposition of coatings on temperature-sensitive materials. It produces uniform and high-purity films with control over the thickness and composition. The reduced heating of the chamber walls minimizes unwanted reactions, leading to fewer impurities in the final coating. The graphene layers obtained using Raman spectroscopy at different parameters of the CW-CVD process were verified, their properties were investigated, and the functional mechanical behavior of the studied graphene-covered stent was confirmed. In vitro, graphene-coated stents promoted rapid endothelial cell repopulation, an advantage over gold-standard drug-eluting stents delaying re-endothelialization. Also, full-range biocompatibility studies on potential allergic, irritation, toxicological, and pyrogenic reactions of new material in vivo on small animal models demonstrated excellent biocompatibility of the graphene-coated stents.

## 1. Introduction

Revascularization of coronary artery stenosis requires stent implantation associated with local vascular endothelial damage, denudation, and potential in-stent clinical complications caused by delayed vascular healing. Hence, developing a stent with a surface that ensures endothelial repopulation while counteracting excessive growth of smooth muscle cells and stent thrombosis is a priority challenge in angioplasty [1,2]. Currently, the most used coronary stents are made of cobalt–chromium (Co-Cr) alloys with a coating of biodegradable polymer that releases a drug, usually from the litmus group (sirolimus, zotarolimus, and everolimus), also called drug-eluting stents (DES). Moreover, special coatings to improve stent properties are being considered. The coatings can be organic (i.e., endothelial cells, antibodies, and their fragments, and cytokines) and inorganic (i.e., oxides, nitrides, silicides and carbides, precious metals, hydroxyapatite-based materials, diamond, and diamond-like carbon). However, they have not shown significant improvements in application results over DES. The surface properties of diamond-like carbon (DLC) and its suitability for medical applications are presented in a review [3]. The material has been found to have the required mechanical properties, surface characteristics, and good biocompatibility [4,5] and has been successfully used as a coating material for medical implants [6]. In vitro results show that DLC and doped-DLC layers can prevent thrombus formation in vascular applications and exhibit good bio- and hemocompatibility [7].

Graphene and graphene-like materials attract great interest due to their unique physical and chemical properties, which have applications in optics, electronics, material science, medicine, etc. [8,9,10,11,12]. Furthermore, in biomedical engineering, graphene-based materials have potential applications in areas such as biosensors and tissue engineering, as well as in creating implants that can better integrate with the body and lower corrosion [13,14,15]. At the same time, considerable attention is paid to studying the cytotoxicity of such materials [16].

The rising interest in the field of graphene research led to the discovery of different synthetic routes allowing to obtain the high-quality layers of graphene: mechanical cleavage, liquid-phase exfoliation, epitaxial growth, chemical vapor deposition (CVD), and chemical cleavage (graphite oxidation followed by chemical reduction of obtained oxide) [17,18,19]. From the abovementioned methods, CVD allows graphene with the most minor surface defects and the highest 2D crystal domain sizes to be obtained [17,20,21]. The CVD method is commonly used to produce graphene on pure metal substrates such as copper or nickel or alloys based on them [22,23]. However, several studies are exhibiting the possibility of obtaining a layer of graphene on metal alloys containing copper, as well as on medical alloys such as stainless steel [24], nitinol [25,26], Ti-based alloys [27], etc. Additionally, a modified cold-wall CVD (CW-CVD) method has been successfully used to obtain a high-quality graphene layer for cobalt [28] and cobalt–nickel alloy [29]. Recently, the possibility of graphene coating on a disc L-605 alloy with CW-CVD [9] was reported.

In this paper, we present the results of optimizing the CW-CVD process previously validated for 2D substrates to a method that enables the effective coating of 3D scaffolds in the form of real stents. Traditional metal or polymer-coated stents are effective but can lead to complications such as restenosis (re-narrowing of the vessel) or inflammatory reactions. Applying graphene, a highly biocompatible material, can reduce the risk of inflammatory responses, thrombosis, and restenosis. This publication presents the research results on efficiently coating 3D mesh implants with a graphene layer. Previous studies on graphene have primarily focused on graphene layers applied to 2D surfaces. The technical challenge was ensuring a uniform coating on a vascular stent with a 3D structure. Furthermore, the increased radial force of the functionalized stent may lead to thinner struts, a current trend in stent engineering. Thus, this made it possible to study, for the first time, the mechanical properties of finished graphene-coated stents (GC-stents) and to confirm that these properties are at least equivalent to stents used in clinical practice. It also made it possible to determine their biocompatibility, which is the final step before conducting large-animal studies and, in the next step, clinical trials. The mechanical properties of the graphene-coated stents have been studied at the macroscopic scale. The resulting GC-stents were subjected to preliminary biocompatibility studies in vitro with endothelial cells and allergic, irritation, toxicological, and pyrogenic in vivo studies in laboratory animals before their planned future implantation into the porcine aorta.

## 2. Results and Discussion

### 2.1. Morphology and Mechanical Properties of the Graphene-Coated Cardiovascular Stents

**Figure 1:**
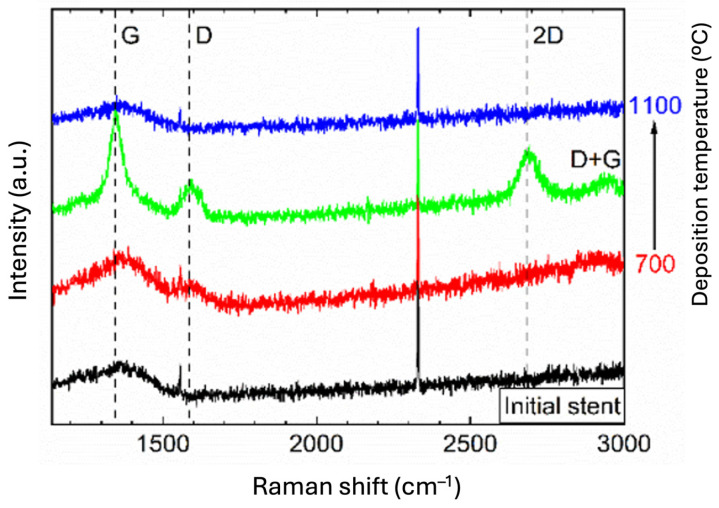
Raman spectra (λ_ex_—514 nm) of the cardiovascular stents before (black line) and after CW-CVD (red, green, and blue line) with different deposition temperatures (700 °C, 900 °C, and 1100 °C, respectively).

The presence of graphene on the stents was confirmed by Raman spectroscopy (Figure 1).

The broadband in the spectra with a maximum at around 1350 cm^−1^ for the initial stents can be explained by the presence of the impurity graphite phase in the non-CVD deposited material. In the spectra recorded after the deposition process, characteristic one-phonon peaks attributed to the G- (~1590 cm^−1^) and D-bands (~1350 cm^−1^) of disordered sp2 carbon appeared, which was best seen for the sample obtained at the optimum deposition temperature of 900 °C. Less intense second-order peaks attributed to 2D (~2710 cm^−1^) and D+G (~2940 cm^−1^) bands were also detected [30,31].

The number of graphene layers was determined from the ratio of 2D to G intensity and was, on average, between 3 and 4 layers for the sample obtained at 900 °C. No areas containing more than 10 graphene layers were observed on the obtained medical devices. There were no more than five layers of graphene on some products. At the same time, the examination showed the heterogeneity of the obtained graphene coating, i.e., it was rough, with different thicknesses ranging from 1 to 4 or up to 5 layers of graphene. Differences in the number of graphene layers should not affect biocompatibility properties, as only the outer layer came into contact with the tissues.

After graphene deposition on the stents, they were checked for functional mechanical characteristics. The testing process consisted of mounting and expanding vascular stents with balloon catheters. After reaching the test endpoint, the specimen was removed from the test apparatus and carefully removed from the mock vessel. Subsequently, reference and graphene-coated stents were analyzed using SEM. The mechanical properties of the stent were adequate despite differences in layer thickness; however, the impact of layer thickness on these properties requires more detailed investigation.

**Figure 2:**
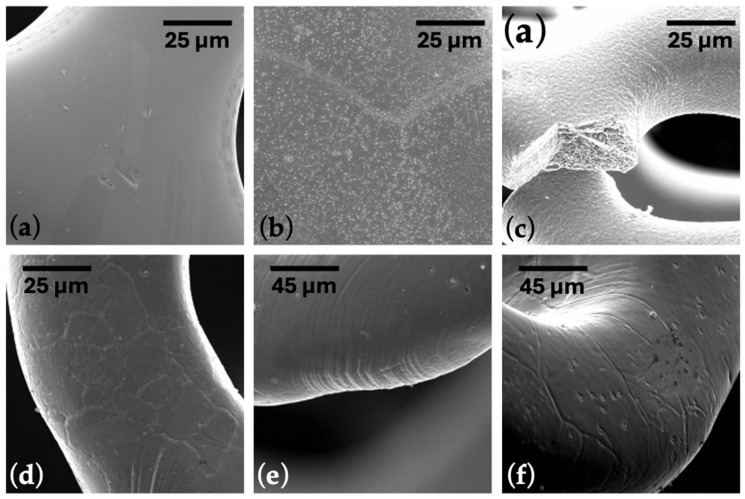
SEM images of a cardiovascular stent (**a**) before and (**b**) after CW-CVD. (**c**) The stent fracture after crimping. (**d**–**f**) Images of critical areas for properly crimped and expanded stent. The scale bar is presented in the appropriate image.

In Figure 2a,b, it is easy to see the difference between the stent surface before and after graphene application. When the stent was crimped to the catheter mounting diameter, cracks in the stent material were observed (Figure 2c).

This results from temperature and probably a hydrogen atmosphere during the CW-CVD process. Given this, the technological process was analyzed, and it was decided to use graphene application before heat treatment. The optimization of the technological process was carried out based on experience in designing and manufacturing stents made of L605 alloy. First, samples of graphene layers were applied to the stents after completing the full production cycle. After observing cracks on the stent, the graphene coating process was conducted before the final annealing of the stents. This optimization can potentially be applied to stents made of other alloys, but annealing process parameters must be appropriately adjusted. The order of technological operations was changed in the following way: laser cutting, electropolishing, graphene deposition, and, in the final step, heat treatment.

**Figure 3:**
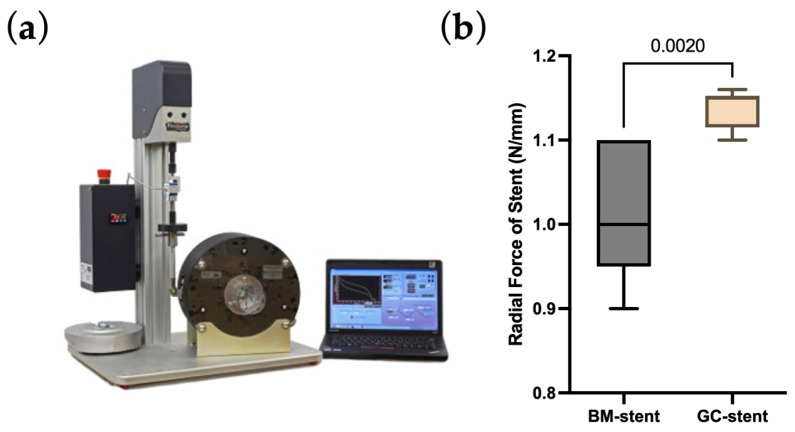
(**a**) The radial force measurement device. (**b**) The values of the obtained radial forces of graphene-coated stent (GC-stent) and uncoated stent (BM-stent). The *p*-value according to the non-parametric Mann–Whitney U test.

Modifying the technological process brought the desired changes, and no cracks were observed for the stents mounted on a balloon mechanical examination (Figure 2d–f). The radial strength of the stent was normal, like that of stents functioning in daily clinical practice. Ramp parameters were used for all samples: cycle speed was 1 mm/min, and stent compression was to half the nominal stent diameter. The average radial force (Table 1, Figure 3) of the stents with graphene was 1.14 (N/mm, 1 mm reference length), 10% higher compared to standard stents, which was 1.02 (N/mm, 1 mm reference length). According to the non-parametric Mann–Whitney U test with a significance level of 0.05, the differences between the radial forces for the BM and GC-stents were statistically significant (*p*-value = 0.0020). The non-parametric approach is due to not meeting the criterion of normality of distribution according to the Shapiro–Wilk normality test with a level significance of 0.05 (*p*-value = 0.0036 for the GC-stent studied group).

**Figure 4:**
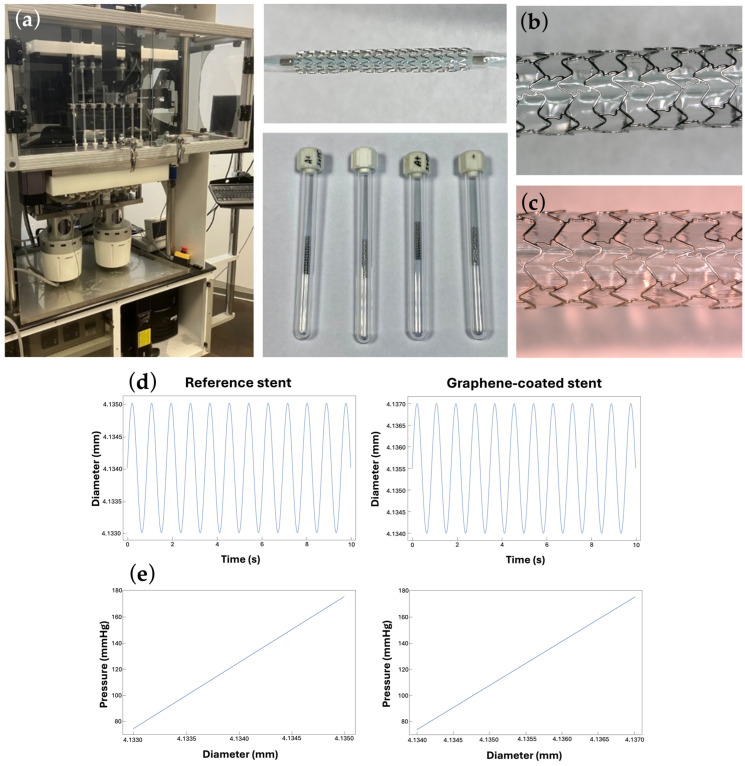
(**a**) The coronary stents used in the test and the BOSE 9400 MAPS system. (**b**) The macro-photography of the bare metal stent. (**c**) The macro-photography of the graphene-coated stent. (**d**) Time evolution of the stent diameter for the reference and graphene-coated stents and (**e**) pressure-diameter elastic behavior of the stent in the cyclic load-unload test for the reference and graphene-coated stents. The response to cyclic loading confirms that graphene-coated stents are just as safe as uncoated stents, which have been used clinically for many years. The mechanical properties of graphene-coated stents are similar to those of other coatings. The main advantage of graphene coating is increased biocompatibility.

Figure 4a shows the laboratory setup and mocking system. Figure 4b,c shows the macro-photographies of the BM-stent and GC-stent, respectively. Figure 4d reports a plot of the diameter variation of the stents in a central period of the test, both for the reference stent and for the coated one. Figure 4e provides the constitutive behavior of the stent during the test.

The presented results for stent diameter evolution (Figure 4d) and elasticity (Figure 4e) were within the tolerance limits for cardiovascular stents. The differences in stent diameter as a function of pressure indicate that graphene-coated stents are minimally more flexible.

### 2.2. The Biocompatibility of Graphene-Coated Stents Towards Endothelial Cells

**Figure 5:**
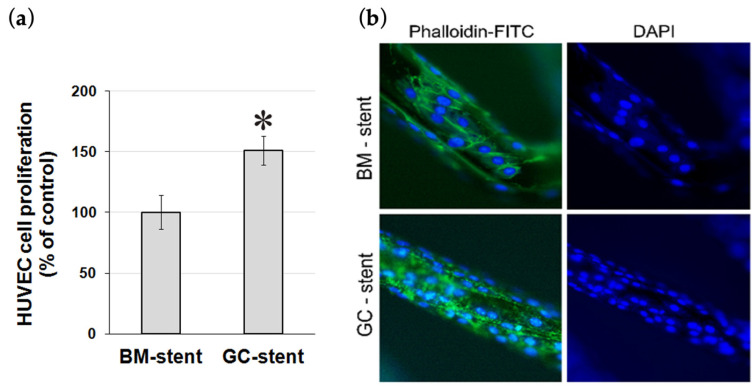
(**a**) HUVEC cell proliferation on bare metal (BM) stents and graphene-coated (GC) bare metal stents after 72 h quantified in the WST-1 assay. * *p* < 0.001. (**b**) The proliferation of HUVEC cells on bare metal (BM) stent and graphene-coated bare metal (GC) stent after 72 h. Cells were visualized by staining the cell’s actin cytoskeleton with phalloidin-FITC and the cell’s nuclei with DAPI. Magnification 400×.

Rapid stent surface re-endothelialization after stent implantation is critical to prevent intimal thickening and vascular thrombosis. Hence, the ability of HUVEC cells to populate the surface of a graphene-coated stent in vitro was studied after 72 h. The results demonstrated that HUVEC cell growth was 51.2% higher (*p* < 0.001) on graphene-coated stents compared to reference stents (i.e., bare metal stents), indicating that the graphene coating supports endothelial cell adherence and proliferation (Figure 5).

These results confirmed our previous study of graphene-coated 316L steel stents, which also better supported the growth of human primary coronary artery endothelial cells (HPCAECs) than stents without a graphene layer [8]. Similar observations were noted by Podila et al. [25], who studied the biocompatibility of graphene coatings on nitinol (Gr-NiTi). According to their study, the graphene coating promoted the growth of endothelial cells and reduced platelet activation, confirming its good biocompatibility. Excellent hemocompatibility of graphene-coated stents was also demonstrated by ELSawy et al. [32]. In their study, the same number of white and red blood cells adhered to graphene-coated stents as non-graphene-coated stents, regardless of blood origin, i.e., healthy controls vs. diabetic patients vs. hypercholesterolemic individuals. Ge et al. [33] reported a study on 316L stents coated with a double layer of graphene oxide that effectively counteracted thrombosis and in-stent restenosis after the implantation into rabbit carotid arteries for 4–12 weeks.

Altogether, these studies indicate that GC-stents have the advantage over DES and polymer stents, which, although it has minimized rates of in-stent restenosis, they still have many disadvantages. Released by DES, immunosuppressive drugs, such as sirolimus and biolimus, inhibit vascular smooth muscle proliferation, arrest the cell cycle, and aid the inhibition of hyperplasia induced by vascular smooth muscle cell proliferation. On the other hand, affecting the cell cycle, these drugs also delay re-endothelialization, induce endothelial dysfunction, and disturb endothelial healing [34,35]. According to Beska et al., DES caused endothelial dysfunction in a long-term study, increasing the risk of recurrent angina following PCI. In turn, biodegradable stents carry the risk of allergic reactions to the polymers used [36,37].

Although few studies have been conducted on graphene-coated stents, the results indicate that the graphene layer provides excellent biocompatibility and hemocompatibility [8,25,32,33]. Previous in vitro and in vivo studies on small laboratory animals suggested that the graphene layer effectively increases endothelial cell proliferation, decreases platelet adhesion, reduces the risk of thrombosis, and minimizes the re-stenosis of arteries.

### 2.3. In Vivo Studies of Graphene-Coated Cardiovascular Stents

**Figure 6:**
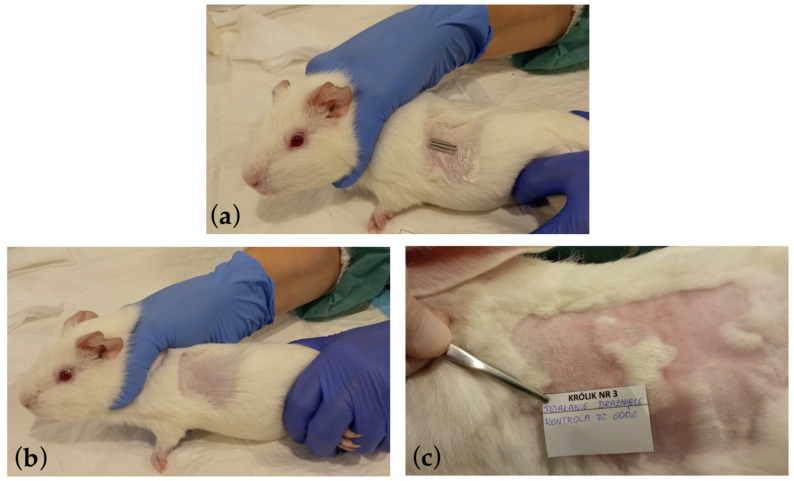
Photographic documentation of the allergy and skin irritation tests performed, where (**a**) the method of applying the tested graphene-coated samples on the shaved skin of a guinea pig during the GPMT test is presented. The tested implant was placed on the skin of a rabbit similarly during the Rabbit Skin Primary Irritation Test. (**b**) The site after applying the graphene-coated stent and after a 14-day break and re-applying of the stent. The site was assessed using the Magnusson and Kligman scale in the GPMT test. (**c**) The site after 72 h where the graphene-coated stent was applied and subjected to erythema and edema assessments on a scale of 0 to 4 in the Rabbit Skin Primary Irritation Test.

**Figure 7:**
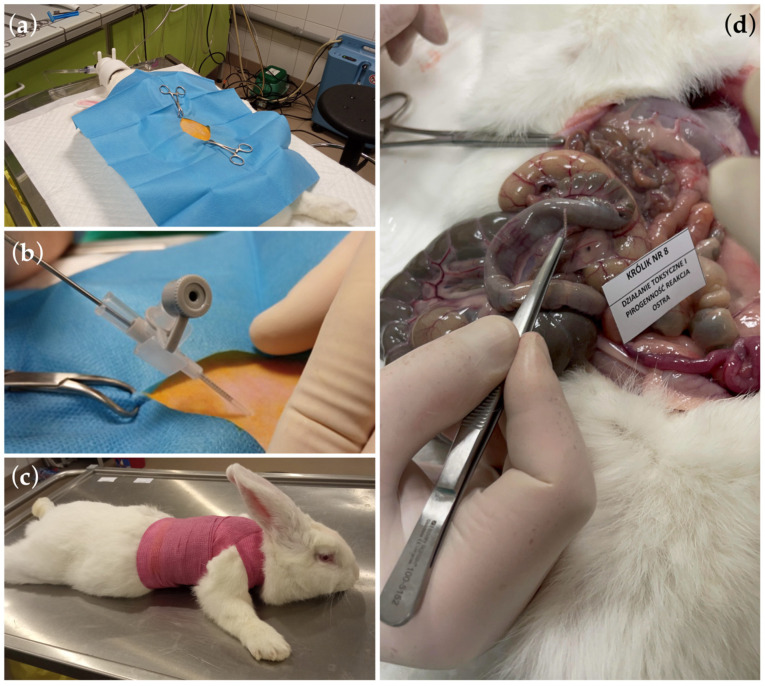
Photographic documentation (**a**–**c**) of individual stages of intraperitoneal insertion of the tested graphene-coated stents. (**d**) The autopsy did not show any symptoms of reaction to the tested material. The implanted material samples were loose in the peritoneal cavity and could be easily removed.

In the conducted study, the in vivo biotoxicity tests of graphene-coated stents on animals were performed, i.e., allergy and skin irritation tests (Figure 6) and systemic toxicity and pyrogenicity tests (Figure 7), enriching the knowledge of graphene coatings biocompatibility on stents.

**Figure 8:**
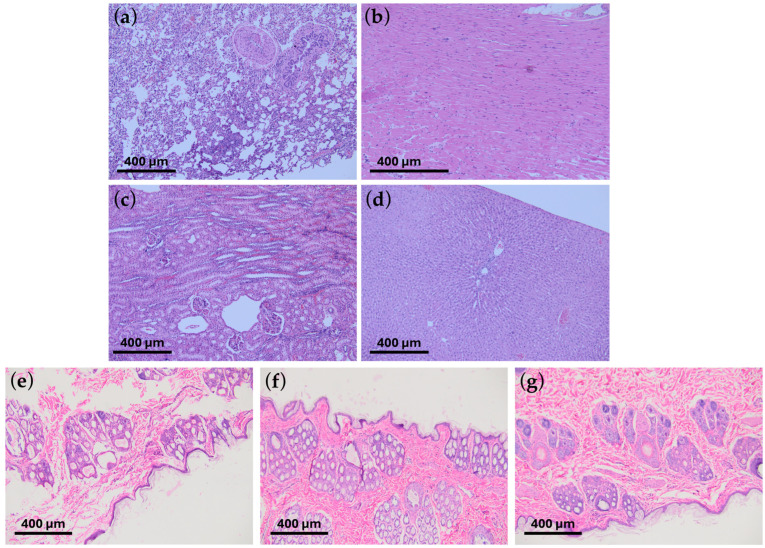
The histopathological microscope images show no organ changes following the introduction of graphene-coated stents: chronic response study. No changes were observed in (**a**) lungs, (**b**) heart, (**c**) kidneys, and (**d**) liver. The results do not differ from typical images characteristic of healthy organs. Below, histopathological images of the skin after (**e**) 24 h, (**f**) 48 h, and (**g**) 72 h, respectively, are shown in the skin irritation tests. The tests were performed on the White New Zealand rabbit. The scale shown in the images indicates 400 μm.

These studies showed that graphene coatings are chemically inert and do not induce allergic or inflammatory reactions in animals. In allergy testing, i.e., the guinea pig maximization test (GPMT), the mean result in the Magnusson and Klinsmann Scale was 0 ± 0 (Figure 6b). In irritation tests, i.e., rabbit primary skin irritation tests, the mean result was 0 ± 0, referred to as “no erythema” (Figure 6c). Rabbit general toxicity and pyrogenicity tests (acute and chronic) showed no adverse effects on surrounding tissues and other organs (Figure 7d and Figure 8).

Throughout the observation period, the animals were characterized by good appetite and activity, and the results of clinical examinations remained within normal limits (Table 2). Also, in the autopsy and histopathological examination, no abnormalities were found.

Based on the results, the excellent biocompatibility of graphene-coated to a Co-Cr stent (L-605) according to ISO 10993 standards in small animal models has been proven. A study by Ge et al. [33] on zebrafish embryos also confirmed the lack of toxicity of graphene oxide. According to their study, graphene oxide coating has no significant effect on the leading physiological indicators such as survival rate, hatching rate, and heart rate of zebrafish embryos.

## 3. Materials and Methods

### 3.1. Graphene Layer Deposition on the Surface of the Cardiovascular Stents

A commercial nanoCVD-8G system (Moorfield Nanotechnology Ltd., Manchester, UK) was used for graphene deposition with process parameters described early elsewhere [9] and summarized in Table 3. Briefly, in the first stage, the heater plate where stents were located was heated to a given temperature (700 °C, 900 °C, and 1100 °C, respectively) in an atmosphere of a mixture of gases (Ar:H_2_—5:2) and kept for one hour. In the next step, graphene was deposed from the gas phase (CH4:H2—1:1). In the last step, the cooling was up to 100 °C in the Ar atmosphere at a set pressure (2 Torr).

### 3.2. Raman Spectroscopy Studies of the Deposited Graphene Layer

Raman spectra were recorded employing a Raman microscope (20× magnification) with a confocal function (Renishaw plc, Wotton-under-Edge, UK). As an excitation source, an Ar laser (514 nm, maximum power on the sample surface ~9 mW) and a CCD camera (Renishaw plc, Wotton-under-Edge, UK) were used as a detector. The detection range was set to 100–3200 cm^−1^.

### 3.3. Mechanical Properties of the Graphene-Coated Stents

Investigation of the mechanical properties of graphene-coated stents has been conducted using a two-scale approach: a macroscale approach, implementing a mocking circulation for 1 million cycles of radial pressure with a specified amplitude and frequency contrasting coated and uncoated stents’ lateral expansion, and a nanoscale approach.

The aim of the point-first approach involves the stent’s mechanical behavior in the presence of high-cycle fatigue at a specified frequency under radial pressure applied with a BOSE MAPS 9400 system. In more detail, the tests must comply with the following codes: ISO 25539-2020, ASTM F2477, and ASTM F2942 [38,39,40]. The stents were subjected to physiologically relevant diametric distension levels using hydrodynamic pulsatile loading, and the pressure range used was 75 to 175 mmHg. Hydrodynamic loading causes a change in the ID of a mock vessel by injecting a volume of fluid into the confined test volume. The OD diameter variations were recorded using a laser device: Keyence LS-7601 Optical/Laser Micrometer Controller (KEYENCE International, Mechelen, Belgium) with Monitor Function. The stents were tested for 518.400 cycles (Physiological Pulse Rate was 1.2 Hz or 72 beats per minute according to ISO 25539-2-2020, for a 5-day test). Radial force was measured on a dedicated BLOCKWISE Radial Force Tester TTR2 with a strain gauge force measurement (BLOCKWISE, Tempe, AZ, USA).

The second approach involves observing the surface nanostructure of the cardiovascular stent subjected to examination. The surface of graphene-coated cardiovascular stents and the surface of reference stents were observed using a Quanta 200 scanning electron microscope (FEI, Hillsboro, OR, USA). The magnification used was 200× to 3000×, and the Wehnelt electrode voltage was 25 kV with a Hi-Vacuum mode.

### 3.4. Graphene-Coated Stents Biocompatibility Assessment with Endothelial Cells

The biocompatibility of the stent was assessed in an in vitro study with a human umbilical vein endothelial cell line (HUVEC; Gibco, Thermo Fisher Scientific, Waltham, MA, USA). HUVEC cells were routinely cultured in Human Large Vessel Endothelial Cell Basal Medium (Gibco, Thermo Fisher Scientific, Waltham, MA, USA) supplemented with Low Serum Growth Supplement (LSGS; Gibco, Thermo Fisher Scientific, Waltham, MA, USA) and 1% penicillin–streptomycin (Gibco, Thermo Fisher Scientific, Waltham, MA, USA). HUVEC cells were cultured at 37 °C in a humid atmosphere with 5% CO_2_ for experiments. The culture medium was changed every second day.

The non-radioactive spectrophotometric WST-1 assay measured HUVEC cell viability and applied proliferation. Sterile stent pieces (BMS, bare metal stent, and GBMS, graphene oxide coated bare metal stents) were placed in the 24-well cell culture plate wells and washed once with phosphate-buffered saline (PBS, pH 7.4) to wet their surface. Then, HUVEC cells were seeded at a density of 1 × 10^5^ cells/mL into the wells and incubated for 24 h. The next day, the stents were transferred into new wells and washed twice to remove loosely bound cells. Then, culture medium was added to the wells, and the samples were incubated for the next two days. After 72 h, WST-1 reagent was added into wells with stents and incubated for two hours to measure cell viability and proliferation. The assay was repeated three times, and the results are the mean with standard deviation. Statistical significance was assessed with a *t*-test, with *p* < 0.05 considered statistically significant. HUVECs growing on stents were visualized on stents after 72 h of incubation under a fluorescence microscope after staining the cell’s actin cytoskeleton with FITC-phalloidin (Invitrogen, Thermo Fisher Scientific, Waltham, MA, USA) and with DAPI (Invitrogen, Thermo Fisher Scientific, Waltham, MA, USA) to visualize cells’ nuclei.

### 3.5. Allergy Tests—Guinea Pig Maximization Test (GPMT)

Allergy tests were performed following the Memorandum Classification and Categorization of Skin Sensitizers and Grading of Test Reactions of the Scientific Committee for Consumer Products (SCCP) on guinea pigs. Dunkin–Hartley guinea pigs were used to assess allergic effects.

The animals were observed for skin lesions 48 h after the initial application of the samples. After a 14-day break, the samples were reapplied, and after another 48 h, skin lesions were assessed according to the Magnusson and Kligman Scale (Table 4). Finally, the animals were euthanized and subjected to histopathological examination.

### 3.6. Irritation Tests—Rabbit Skin Primary Irritation Test

According to the recommendations of ISO 10993-11:2017 [41], the skin reaction through irritation tests (i.e., acute primary irritant dermatitis) was assessed. This is a part of the Biological Evaluation of Medical Devices standards, specifically Part 11. A study on systemic toxicity was conducted on a White New Zealand rabbit, a commonly used animal model for irritant evaluation. On the eve of applying the test material, the fur was shaved from the left side of the body. The 0.5 cm^2^ plaque was attached to the skin with a patch on the left side of the shaved field. The area on the right served as control. An assessment was repeated during the study after 24, 48, and 72 h. Additionally, an anatomopathological examination was performed 14 days after the material removal.

The evaluation included erythema and edema assessments on a scale of 0 to 4, e.g., 0 no redness, 4 severe redness.

### 3.7. Toxicological and Pyrogenic Studies—Rabbit Toxicity Studies and Pyrogenicity Studies

Systemic toxicity and pyrogenicity studies were performed following the recommendations of ISO 10993-11:2017—Biological Evaluation of Medical Devices—Part 11. Toxicity and pyrogenicity studies were performed in rabbits as the species of choice for such studies. After implantation in the form of an intraperitoneal injection of a material sample 1 cm long and 0.9 mm in diameter, the acute reaction was assessed up to 72 h after implantation and the chronic response up to 6 months after implantation. Both reactions were performed on 10 animals. During the follow-up period, animals were clinically examined weekly, including core body temperature, pulse rate, and respiration. Immediately before euthanasia, blood was taken from the animals for hematological and biochemical tests (liver and kidney tests to assess the acute reaction and electrolytes and carbohydrates for the chronic reaction). After euthanasia and post-mortem examination, biopsies were taken for histopathological examination. These studies evaluated the response of tissues and organs to the implanted material.

## 4. Conclusions

The study confirmed the possibility of applying graphene to the surfaces of a three-dimensional implant (i.e., cardiovascular stent). Functional testing of the stents after the CW-CVD process revealed a severe problem: a change in the substrate structure and cracking during testing. This was the effect of temperature and probably the hydrogen atmosphere during the CW-CVD process. It is shown that only the introduction of pre-annealing of the L605 alloy stent makes it possible to achieve the effect of covering its surface with a graphene layer in the CW-CVD process. The stent’s functional and radial force tests confirmed that appropriate mechanical parameters were achieved. The observed stents show a completely elastic behavior in the physiological pressure range chosen for the tests. Still, a singular feature is related to the maximum diameter extension of the stent. Indeed, in the test, a larger value of the diameter expansion under the superimposed pressure cycle was observed for the coated stent compared to the uncoated stent. This surprising behavior is observed for an additional radial strain of 11% for the uncoated stent. Observation of the surface stent has shown a bunch of micro-cracks in the stent coating that may be due to the expansion phase that may have also involved the surface of the strut influencing their stiffness in the elastic range, justifying the increment in the radial strain observed. Additional tests to confirm such a hypothesis and the influence on the fatigue strength of the stent must be performed. However, the result of the study showed, preliminarily, that the mechanical performance of the coated stent is very similar to the commercially available one in terms of fatigue-life strength and that a slight decrement in the device stiffness is observed. Therefore, using the coated stent may be convenient as more device compliance is required for the vessel. The ability of graphene-coated stents to support re-endothelialization, thereby inhibiting thrombosis. Further, the lack of toxicity for small laboratory animals indicated in our study authorizes us to conduct further preclinical long-term studies on small animals and study the implementation of graphene-coated stents into the porcine aorta.

## Figures and Tables

**Table 1:** The statistical parameters of the radial force of tested stents. (*) According to the non-parametric Mann–Whitney U test.

	Mean	SD	Median	IQR	*p*-Value
BM-stent	1.020	0.084	1.000	0.150	0.0020 *
GC-stent	1.139	0.024	1.115	0.038	

**Table 2:** Blood morphology and biochemistry results were determined at the indicated time points. All values are within reference ranges for White New Zealand rabbits.

	24 h	48 h	72 h	6 Months
RBC (10^6^/μL)	5.60 ± 0.47	5.59 ± 0.50	5.65 ± 0.42	6.15 ± 0.30
Hb (g/dL)	11.78 ± 0.97	10.85 ± 3.32	12.13 ± 0.96	13.87 ± 0.76
HCT (%)	34.38 ± 2.91	34.49 ± 3.29	36.21 ± 2.85	37.03 ± 1.95
WBC (10^3^/μL)	6.90 ± 1.60	8.17 ± 3.25	6.92 ± 3.31	6.88 ± 1.55
Urea (mmol/L)	6.03 ± 0.89	5.69 ± 1.12	6.28 ± 0.85	5.90 ± 0.47
Creatinine (μmol/L)	95.72 ± 10.77	79.26 ± 39.35	97.56 ± 9.47	117.90 ± 13.63
AST (U/L)	20.39 ± 6.50	36.10 ± 35.37	19.43 ± 6.02	33.18 ± 8.30
ALT (U/L)	22.56 ± 11.03	26.69 ± 12.80	23.60 ± 10.82	35.28 ± 7.96
ALP (U/L)	69.63 ± 17.45	68.09 ± 23.94	81.89 ± 21.75	53.18 ± 7.40

**Table 3:** The process parameters of CW-CVD.

	t, s	T, °C	P, Torr	Ar, % (sccm)	H_2_, % (sccm)	CH_4_, % (sccm)
SP 1	0	103	+7	5 (100)	1 (20)	0 (0)
SP 2	300	214	+9	5 (100)	1 (20)	0 (0)
SP 3	0	120	+77	5 (100)	1 (20)	0 (0)
SP 4	2700	121	+13	0 (0)	2 (1.2)	35 (20)

**Table 4:** The Magnusson and Kligman scale.

Path Test Reaction	Grading Scale
No visible changes	0
Discrete or patchy erythema	1
Moderate and confluent erythema	2
Intense erythema and swelling	3

## Data Availability

The data supporting this study’s findings are available from the corresponding author upon reasonable request.

## Abbreviations

BMS	Bare Metal Stent
BM-	Bare Metal
CW-CVD	Cold-Wall Chemical Vapor Deposition
DES	Drug-Eluting Stent
DLC	Diamond-Like Carbon
GC-	Graphene-Coated
PCI	Percutaneous Coronary Intervention
SEM	Scanning Electron Microscopy
